# How context links to best practice use in long-term care homes: a mixed methods study

**DOI:** 10.1186/s43058-024-00600-0

**Published:** 2024-06-07

**Authors:** Yinfei Duan, Jing Wang, Holly J. Lanham, Whitney Berta, Stephanie A. Chamberlain, Matthias Hoben, Katharina Choroschun, Alba Iaconi, Yuting Song, Janelle Santos Perez, Shovana Shrestha, Anna Beeber, Ruth A. Anderson, Leslie Hayduk, Greta G. Cummings, Peter G. Norton, Carole A. Estabrooks

**Affiliations:** 1 Faculty of Nursing, College of Health Sciences, University of Alberta, Edmonton, AB Canada; 2 Nursing Department, College of Health and Human Services, University of New Hampshire, Durham, NH USA; 3 Joe R. & Teresa Lozano Long School of Medicine, University of Texas Health, San Antonio, TX USA; 4 Institute of Health Policy, Management and Evaluation, University of Toronto, Toronto, ON Canada; 5 School of Health Policy and Management, Faculty of Health, York University, Toronto, ON Canada; 6 School of Public Health, Bielefeld University, Bielefeld, Germany; 7 School of Nursing, Qingdao University, Qingdao, Shandong China; 8 School of Nursing, University of North Carolina at Chapel Hill, Chapel Hill, NC USA; 9 School of Nursing, Johns Hopkins University, Baltimore, MD USA; 10 Sociology Department, Faculty of Arts, University of Alberta, Edmonton, AB Canada; 11 Department of Family Medicine, University of Calgary, Calgary, AB Canada

**Keywords:** Care aides, Best practice use, Coincidence analysis, Configurational comparative methods, Context, Evidence-based practice, Long-term care, Nursing homes, Research utilization

## Abstract

**Background:**

Context (work environment) plays a crucial role in implementing evidence-based best practices within health care settings. Context is multi-faceted and its complex relationship with best practice use by care aides in long-term care (LTC) homes are understudied. This study used an innovative approach to investigate how context elements interrelate and influence best practice use by LTC care aides.

**Methods:**

In this secondary analysis study, we combined coincidence analysis (a configurational comparative method) and qualitative analysis to examine data collected through the Translating Research in Elder Care (TREC) program. Coincidence analysis of clinical microsystem (care unit)-level data aggregated from a survey of 1,506 care aides across 36 Canadian LTC homes identified configurations (paths) of context elements linked consistently to care aides’ best practices use, measured with a scale of conceptual research use (CRU). Qualitative analysis of ethnographic case study data from 3 LTC homes (co-occurring with the survey) further informed interpretation of the configurations.

**Results:**

Three paths led to very high CRU at the care unit level: very high leadership; frequent use of educational materials; or a combination of very high social capital (teamwork) and frequent communication between care aides and clinical educators or specialists. Conversely, 2 paths led to very low CRU, consisting of 3 context elements related to unfavorable conditions in relationships, resources, and formal learning opportunities. Our qualitative analysis provided insights into how specific context elements served as facilitators or barriers for best practices. This qualitative exploration was especially helpful in understanding 2 of the paths, illustrating the pivotal role of leadership and the function of teamwork in mitigating the negative impact of time constraints.

**Conclusions:**

Our study deepens understanding of the complex interrelationships between context elements and their impact on the implementation of best practices in LTC homes. The findings underscore that there is no singular, universal bundle of context-related elements that enhance or hinder best practice use in LTC homes.

**Supplementary Information:**

The online version contains supplementary material available at 10.1186/s43058-024-00600-0.

Contributions to the Literature

- By integrating configurational and qualitative methods, we achieved comprehensive insight into the complex relationship between organizational context and the use of best practices by care aides in long-term care homes.
- Individual context elements generally did not influence best practice use in isolation. They work together to establish distinct contextual patterns or configurations that were linked to best practice use.
- Context elements such as teamwork and regular communication between care aides and clinical educators or specialists collectively affected best practice use. The presence of effective leadership from care aides’ supervisors alone could be sufficient to enhance best practice use.

## Background

Implementing evidence-based best practices is important to provide optimal care for older adults in long-term care (LTC) homes [1–3]. Although there is an increase in literature on this topic, little research has focused specifically on use of best practices by care aides in LTC homes [1–3], despite the fact that unregulated care aides are the largest workforce in LTC. Care aides provide up to 90% of direct care and social support to residents [4–6]. Beyond support and assistance, care aide responsibilities include keeping up with new knowledge, skills, and best practices to meet the complex care needs of residents [4–6]. 

Context, broadly defined as the environment or setting in which a proposed change is to be implemented [7], is an integral and influential factor in the implementation of best practices [8]. Despite its significance to best practice use, and other workplace behaviors, context has no universally accepted definition. Various key elements of context are proposed by widely used frameworks such as the Promoting Action on Research Implementation in Health Services (PARIHS) framework and the Consolidated Framework for Implementation Research (CFIR) [7, 9, 10]. These elements include organizational support, financial resources, social relationships and support, leadership, as well as organizational culture and climate [8]. These frameworks depict context at varying levels of aggregation, from macro (beyond the organization), through meso (organizational level), to micro (local unit, team, or group) [8]. 

Empirical research consistently indicates a significant relationship between context and best practice use across health care settings [2, 11]. Studies have focused primarily on meso (organizational) level context [11]. A systematic review by Li et al. highlighted six commonly reported elements of organizational context that influence implementation of best practices across a range of health care settings [11]. These include organizational culture, leadership, networks and communication, resources, feedback mechanisms, and champions. Findings were later corroborated in a systematic review of qualitative studies focusing on LTC settings by McArthur et al. [2] They identified common barriers to best practice use—time constraints, inadequate staffing, lack of resources, lack of training, and lack of teamwork and organizational support. Conversely, leadership, the presence of champions, well-designed strategies, protocols, resources, and time were frequently identified as facilitators. [2].

### Complex relationships between organizational context and best practice use

To move beyond simply identifying individual elements of organizational context linked to best practice use, it is necessary to explore how these elements interact. Both theoretical and empirical studies report that context elements are interconnected, exerting their influence in complex and dynamic ways [2, 11–22]. Such complexity and dynamism are integral to complex adaptive systems where implementation efforts occur [15, 23–26], such as LTC homes. Like in other health care settings, various agents (e.g., residents, their families, care aides, nurses, doctors, administrative staff) interact with each other and their surroundings [15, 23–25]. These interactions stimulate self-organization, leading to the emergence of behaviors and practices that adapt to the system’s unique challenges [15, 23–25]. This reality underscores the absence of a single, universal configuration of context elements contributing to implementation outcomes. The complexity and diversity underscore the value of investigating the interplay among context elements, rather than studying them in isolation [15, 24, 25, 27]. 

Exploring complexity embedded in contextual relationships presents methodological challenges [27–29]. Researchers often use qualitative methods, regression-based quantitative methods, or both, when studying complex relationships between context and implementation outcomes [12, 13, 30, 31]. While these methods, whether used individually or in combination, offer valuable insights, they might be less optimal for investigating questions designed to identify patterns of elements that affect specific outcomes (i.e., complex configurational questions) [31–33]. Qualitative methods often prioritize depth over breadth, which could sometimes mean fewer cases are explored in detail rather than systematically comparing a large set. Regression-based quantitative methods, while used for examining interactions [12, 13, 30], focus on average effects, holding other factors constant, which do not adequately represent real world complexity [31]. Testing all possible interactions or even many, is not usually feasible due to challenges such as insufficient sample sizes and multiple testing issues [31–33]. 

The application of configurational comparative methods in implementation research has provided another tool for studying complex questions [31–36]. These methods use mathematical set theory and Boolean algebra to identify combinations of explanatory factors that distinguish one group of cases from another in relation to specific outcomes [31–34]. These methods offer a holistic case-based view, akin to qualitative research, while also generating quantified cross-case findings similar to quantitative analysis [31–34]. Despite growing use in implementation and health services research, configurational comparative methods in combination with other methods is yet to be explored in LTC research. This study directly addresses this gap in using configurational approaches to answer complex questions about the relationships between context and best practices use within LTC settings.

### Study purpose and aims

Our study sought to develop comprehensive and more nuanced understandings of context elements and their interrelationships associated with use of best practices by care aides in LTC homes, using an innovative mixed methods approach that combines configurational and qualitative methods. Our research aims were to: (1) identify clinical microsystem (care unit)-level contextual configurations linked to best practice use and (2) understand the role of individual context elements and their combinations (forming contextual configurations) that influence best practice use.

**I**n our study, the outcome ***use of best practices*** was specifically defined as ***conceptual use of best practices***. This involves critically reflecting on research-based knowledge and applying it to inform clinical decision-making [37–39]. We differentiated conceptual use from instrumental use of best practices, the latter referring to the direct application of codified and explicit evidence-based knowledge, such as standardized protocols. [30, 37–40].

## Methods

### Study design

This study is situated within a large parent study, the Influence of Context on Implementation and Improvement (ICII), that focused on the analysis of over 15 years of secondary data collected through the Translating Research in Elder Care (TREC) program [41]. ICII was designed as a mixed methods study; it dedicated significant resources to convene experts in quantitative and qualitative data analysis techniques to generate theoretical and practical implications for promoting implementation and improvement success [41]. This study is reported following the Mixed Methods Article Reporting Standards (MMARS, see Additional File 5) [42].

Our analysis combined configurational and qualitative methods. Specifically, we conducted a coincidence analysis (a type of configurational comparative analysis) of the quantitative survey data and a directed content analysis of the qualitative case study data. The collection of both quantitative survey data and qualitative case study data occurred concurrently during an early wave of TREC data collection in 2008–2009. This was the only wave of data collection where coincident quantitative and qualitative data were available for our use, providing a unique opportunity for this study.

Findings from each method were integrated at reporting and interpretation stages. We focused on clinical microsystem (care unit)-level context. A clinical microsystem represents an assigned group of individuals working together regularly to provide care to specific subsets of patients, while sharing common aims, processes, information, and outcomes [43–46]. They are critical components for the production of quality in any organization [43–46]. 

### Data sources, setting, and sample

Data for coincidence analysis were obtained from the survey data of 1,506 care aides working in 103 care units within 36 LTC homes in 3 Canadian prairie provinces (Alberta, Saskatchewan, and Manitoba). Participating LTC homes included 30 LTC homes from urban areas across the provinces, selected using a stratified random sampling based on region, LTC home ownership model, and home size. Additionally, a convenience sample of 6 LTC homes was chosen from rural areas in Saskatchewan. Care aides working in participating LTC homes were invited to complete a survey through in-person structured interviews if they met eligibility criteria (i.e., working on the care unit for 3 or more months with a minimum of 6 shifts per month). The original TREC survey protocol is reported elsewhere [47]. 

The qualitative data were obtained from TREC case studies conducted in 3 LTC homes—one home from each province that participated in the TREC survey during the same period of data collection [48]. Discussions with health authority decision makers identified the most typical LTC home in each province, based on key characteristics such as size, owner-operator model, types of care units [48]. A case was defined as an individual LTC home and its embedded care units [48]. The original TREC case study protocol and reports of study findings are reported elsewhere [21, 48, 49]. 

Qualitative data comprised written fieldnotes from ethnographic observations and transcripts from in-person interviews. In the original study, a research associate was assigned to a specific LTC home to conduct non-participant observations across all shifts and days of the week and focused on the general flow of events and operations in common areas of the LTC home. Several staff were “shadowed” during their shift, which allowed for detailed exploration of work practices and use of knowledge in care provision, and provided the opportunity for informal discussions with care staff. The research associate documented the observations and their reflections on observations in fieldnotes. Observational data collection took place over 6 months and ended when the data were saturated [21, 48, 49]. The observational data described daily work patterns that provided the organizational context for knowledge use [21, 48, 49]. 

Following the initial analysis of the observational data, semi-structured interviews were conducted with a purposefully selected sample of care staff that covered the following staff categories: care aides, registered nurses (RNs), licensed practical nurses (LPNs), administrators, directors of care, managers, allied health professionals, and practice specialists [21, 48, 49]. In the interviews, participants were asked to describe their roles and responsibilities, their work environment (context), as well as how their work environment could influence the implementation of best practices and innovations [21, 48, 49]. 

### Coincidence analysis

Coincidence analysis identifies a “minimal theory” (i.e., difference-making combinations of conditions that uniquely distinguish one group of cases from another group in relation to a specific outcome) [33, 35, 36]. The analytic objective of coincidence analysis is to search for necessary and sufficient conditions for the outcome to occur [33, 50]. Coincidence analysis addresses causal complexity (when several conditions must jointly appear for an outcome to occur) and equifinality (when multiple paths lead to the same outcome) [33, 35, 36].

### Variables

#### Outcome variable

In the TREC care aide survey, a validated 5-item scale was used to measure conceptual use of best practices or conceptual research use (CRU) (see Table 1) [37].

**Table 1:** Measures of context and conceptual research use in the TREC care aide survey

Variable	Description	No. of items	Scaling scoring	Sample item
Outcome				
Care aides’ conceptual research use	Thinking about research-based knowledge and then using it to inform clinical decision making	5	Mean of the 5 items (range = 1–5) measured on a 5-level scale (from never to almost always)	On your last typical workday on your unit, how often did best practice knowledge about things like pain management, managing difficult behaviors, and managing pressure ulcers help to change your mind about how to care for residents?
**Perceptions of work environment (context) measured with the Alberta Context Tool (ACT)**				
Leadership^a^	The actions of care aides’ supervisors on the unit to influence excellence in practice. The items specifically focus on resonate leadership that emphasizes emotional support, relationship building, and motivation.	6	Mean of the 6 items (range = 1–5) measured on a 5-level scale (from strongly disagree to strongly agree)	The person who I report to most of the time (choose from managers, RNs, or LPNs) looks for feedback even when it is difficult to hear.
Culture^a^	The way that “we do things” on the unit; items generally reflect a supportive work culture.	6	Mean of the 6 items (range = 1–5) measured on a 5-level scale (from strongly disagree to strongly agree)	I receive recognition from others about my work.
Evaluation^a^	The process of using data to assess team performances and to achieve outcomes on the unit.	6	Mean of the 6 items (range = 1–5) measured on a 5-level scale (from strongly disagree to strongly agree)	I routinely receive information on my team’s performance.
Social capital^a^	Active connections among people (bonding, bridging, and linking); specifically, the extent to which care aides freely exchange information about resident care within their work team and between teams and support each other in the job.	6	Mean of the 6 items (range = 1–5) measured on a 5-level scale (from strongly disagree to strongly agree)	People on the team share information with others on the team.
Organizational Slack - Staffing^a^	The cushion of actual or potential staffing resources which allows an organization to adapt successfully to internal pressures; specifically, sufficient staff for care aides to deliver best possible care.	3	Mean of the 3 items (range = 1–5) measured on a 5-level scale (from strongly disagree to strongly agree)	We have enough staff to deliver the best possible care.
Organizational Slack - Space^a^	The cushion of actual or potential resources related to physical space which allows an organization to adapt successfully to internal pressures; specifically, the accessibility of private space for staff to discuss resident care.	2	Mean of the 2 items (range = 1–5) measured on a 5-level scale (from strongly disagree to strongly agree)	We have private space such as a conference room on this unit to discuss resident care and share knowledge about resident care and best practices.
Organizational Slack - Time^a^	The cushion of actual or potential time resources which allows an organization to adapt successfully to internal pressures; specifically, time resources which allows care aides to do extra things for residents and think about best practices.	4	Mean of the 4 items (range = 1–5) measured on a 5-level scale (from strongly disagree to strongly agree)	We have time to do something extra for residents.
Formal interactions^b^	Formal exchanges that occur between care aides and other staff through scheduled activities (e.g., team meetings, change of shirt report).	4	Each item was measured on 5-level scale (never-almost always). Items are recoded to 0 = no interaction (never or rarely), 0.5 = occasionally, 1 = interaction ( frequently, almost always). Take sum of recoded items (range = 0–4).	In the last typical month, how often did you participate in: team meetings about resident care, family conferences, change of shift report, continuing education held outside of your facility.
Informal interactions^b^	Information exchanges that occur between individuals within an organization or unit which can promote the transfer of knowledge.	9	Each item was measured on 5-level scale (never-almost always). Items are recoded to 0 = no interaction (never or rarely), 0.5 = occasionally, 1 = interaction ( frequently, almost always). Take sum of recoded items (range = 0–9).	In the last typical month, how often did you talk with people in the following roles or situations about resident care: other care aides, LPNs, RNs, physicians, other health care providers, any clinical educator/instructor/specialist, someone who brings new ideas to the unit, “hallway talk”, and informal bedside teaching sessions.
Structural resources^b^	The structural elements of an organization that facilitate the ability to assess and use knowledge (e.g., notice boards, journals in the work area, policies and procedure manuals)	7	Each item was measured on a 5-level scale (never-almost always). Items are recoded to 0 = no interaction (never or rarely), 0.5=occasionally, 1 = interaction (frequently, almost always) Take sum of recoded items (range = 0–7).	On your unit, in the last typical month, how often did you use: clinical practice guidelines, policy and procedure manual for your facility, a library, textbooks in your work area, journals in your work area, notice boards in you work area, in-service/workshops/course in your facility.

*RN* registered nurses, *LPN*  licensed practical nurses

^a^Scaled concept

^b^Non-scaled or count-based concept

#### Context variables

The Alberta Context Tool (ACT) was used to measure microsystem-level (or care unit) context as perceived by care aides. The ACT comprises 7 scaled concepts (leadership, culture, evaluation, social capital, organizational slack in time, staffing, and space) and 3 non-scaled, or count-based, concepts (formal interactions, informal interactions, and structural and electronic resources) [51]. Original context measures of the ACT are presented in Table 1.

In our analysis, we strategically selected 4 scaled concepts measured in the ACT: leadership, social capital, organizational slack in time, and organizational slack in space. In the ACT, the Leadership scale measures the actions of care aides’ supervisors on the unit to influence excellence in practice; the items specifically focus on resonate leadership that emphasizes emotional support, relationship building, and motivation [51]. The Social Capital scale captures the extent to which care aides can freely exchange information about resident care within their work team and between teams and support each other in the job [51]. The scale of Organizational Slack in Time measures the cushion of actual or potential time resources which allows care aides to do extra things for residents and think about best practices [51]. The scale of Organizational Slack in Space measures the accessibility of private space for staff to discuss resident care [51]. 

We excluded 3 other scaled concepts (culture, evaluation, and organizational slack in staffing) due to their conceptual overlap with the included concepts. This was to maximize data diversity while maintaining a parsimonious set of conditions (context variables) relative to the number of cases—a requirement for effective coincidence analysis. This is important because as more conditions are added, the number of logically plausible configurations (combinations of all possible conditions) increases exponentially [33]. 

We also created 4 context “meta-factors” from 8 individual items selected from the non-scaled concepts. These items were selected because they are substantively relevant to research use [11–13]. We created the meta-factors by combining items that reflect similar context conditions—a common approach in coincidence analysis to reduce the number of conditions without eliminating either of the properties represented by these conditions [33]. 

Specifically, we combined the items “care aides attend continuing education out of facility” and “care aides attend in-services in the facility” to make a meta-factor *educational activities* (care aides frequently or always attend continuing education or in-services off or on site). The other 3 context meta-factors included *educational material* (care aides frequently or always use policy, procedure manuals, or clinical guidelines), *communication* *with dedicated facilitator roles* (care aides frequently or always talk with educators, clinical specialists, or someone bringing new ideas to the care unit), and *formal meetings* about care (care aides frequently or always participate in team meeting or family conferences).

### Data calibration and analysis

To prepare data for coincidence analysis, we followed a 3-step data calibration process recommended by Whitaker et al. [33] We: (1) aggregated individual-level, care aide-reported scores to the care unit level; (2) categorized care unit-level continuous variables to generate binary care unit properties; and (3) selected a final set of cases (care units) for analysis. These processes are detailed in Table 2.

**Table 2:** Data calibration and data analysis processes for coincidence analysis

Data Calibration Calibrate values of each variable and select cases to create a dataset for analysis	
**Step 1: Data aggregation**	We aggregated individual-level, care-aide-reported scores of the original variables to the care-unit level. The aggregated care-unit-level variables were presented as continuous variables. Specifically, they were expressed as “the proportion of care aides on a given care unit that report scores for the original variable exceeding a certain threshold”. One outcome variable and 8 context variables were selected for coincidence analysis. Additional File 1 presents box plots for the care-unit-level continuous variables. • For CRU, leadership, social capital, organizational slack in space, and organizational slack in time, the care-unit-level scores were expressed as “the proportion of care aides on the unit that reported the scale score equal to or above the median of care-aide-reported scores in the care aide sample”. • For the four context meta-factors (each of which was derived from combing two items), the care-unit-level scores were expressed as “the proportion of care aides on the unit that reported ‘frequently’ or ‘almost always’ for any of the items”. Note that in this step, we excluded 7 care units with less than 8 care aide participants to ensure reliable aggregated care-unit-level variables.
**Step 2: Data categorization**	We categorized the care-unit-level continuous variables to generate categorical or binary care-unit variables. Additional File 2 shows the care-unit-level categorical variables. • For all care-unit-level continuous variables (except for two variables indicated below), we used a quartile-split method to categorize each variable into 4 levels: less than the lower quartile, from lower quartile to median, from median to upper quartile, and equal to or greater than the upper quartile. These categories represent very low, moderately low, moderately high, and very high levels of the care-unit-level continuous variable relative to other care units in the sample. • For two context meta-factors—communication with dedicated facilitator roles and formal meetings about care, we used a median-split method to categorize each variable into two levels: less than the median, equal to or greater than the median. These categories represent low and high levels of the care-unit-level continuous variable relative to other care units in the sample. We used median-split instead of quartile-split for these two care-unit-level continuous variables due to a small range in their distributions.
**Step 3: Case selection**^**a**^	We eliminated the middle set of cases (i.e., care units that fell into the middle two categories of care-unit-level CRU: “from lower quartile to median” and “from median to upper quartile”). This step ensured a substantive difference between the *very high* CRU group (i.e., equal to or greater than the upper quartile) and the *very low* CRU group (i.e., less than the lower quartile). This method has been commonly used in coincidence analysis to build in a meaningful difference between levels of the outcome.^34,35^
**Data Analysis** Perform coincidence analysis using R’s *cna* and *frscore* packages	
Analytic sample included 47 care units (22 with *very high* CRU and 27 with *very low* CRU). Table 3 shows the final set of care unit-level binary variables used for coincidence analysis.	
**Step 1: Identify minimally sufficient conditions**	We set the outcome as *very high* CRU. We used the “minimally sufficient conditions” (i.e., “msc”) function within the *cna* package to identify specific configurations of context variables with especially strong connections to *very high* CRU at five different consistency levels (0.75–0.95 with an interval of 0.05).^49,50^ This process enabled us to further select a parsimonious subset of context variables to include in the model. As a result, we excluded educational activities which did not appear in the configurations with “best of class” coverage scores (i.e., the top coverage scores among configurations with the same number of conditions).
**Step 2: Refine model inputs by recalibrating variables** ^**a**^	Upon reviewing the results of the minimally sufficient conditions generated in Step 1 in Data Analysis, we observed that those quartile-split care-unit-level context variables were more likely to connect to *very high* CRU when they took the highest value (equal to or greater than the upper quartile). Accordingly, we recoded those quartile-split care-unit-level context variables to binary variables by grouping the three lower categories into one category (lower than the upper quartile). This step ensured a list of context variables that was parsimonious relative to the number of cases. The distributions of the original individual-level, care-aide-reported scores of the context variables (which were negatively skewed) also support this recoding process as our intention was to separate care units with the highest-level context scores from other care units with generally high context scores.
**Step 3: Build the final model for the outcome =** ***very high*** **CRU**	We built coincidence analysis models based on the recoded data and used fitness-robustness scores for model selection. Specifically, we used the “rean_cna” function within the *frscore* package to build multiple coincidence analysis models at systematically varied consistency and coverage thresholds and we subsequently used the “frsocre” function to calculate a fit-robustness score for each solution generated by the coincidence analysis modeling process. A fit-robustness score for a solution quantifies the degree to which the solution agrees with other solutions inferred from the same data under the same coincidence analysis modeling process.^48^ We used the “submodel score type” to calculate fit-robustness scores, a method that favors more complex models.^48^
**Step 4: Build the final model for the outcome =** ***very low*** **CRU**	We repeated Step 3 in Data Analysis after changing the outcome variable to *very low* CRU. The purpose was to gain additional insights into the complex context-CRU relationship, because context configurations that contribute to *very high* CRU may not be the reverse of the configurations that contribute to *very low* CRU.

^a^We also performed sensitivity analyses while selecting cases (Step 3: Case selection in Data Calibration) and recoding context variables (Step 2: Refine model inputs by recalibrating variables in Data Analysis). This was done to investigate whether alternative methods for case selection and recoding of context variables might prove more suitable than the initially applied methods. The results of sensitivity analysis supported our initially applied methods (see Additional File 3)

We used R’s *cna* [52] and *frscore* [53] packages for coincidence analysis model building and model selection based on a crisp data set. The final set of care unit-level binary variables included 1 outcome variable (CRU, conceptual research use) and 7 context variables (see Table 3). In coincidence analysis, 2 important indices evaluate model performance: consistency and coverage. *Consistency* is the proportion of cases exhibiting a certain path (also referred to as solution or configuration) that yields the specified outcome; *coverage* is the proportion of cases having the specified outcome that are explained by any identified path [54, 55]. Table 2 describes our 3-step analysis.

**Table 3:** Final set of care-unit-level binary variables used for coincidence analysis

Variable	Coding and interpretation
**Outcome** Conceptual research use (CRU)	A care unit was assigned a value of 1 if the proportion of care aides reporting CRU > = 4^a^ was equal to or greater than the upper quartile among all care units in the sample. This means that the care-unit-level CRU is *very high* compared to other care units in the sample. A care unit was assigned a value of 0 if the proportion of care aides reporting CRU > = 4^a^ was less than the lower quartile among all care units in the sample. This means that the care-unit-level CRU is *very low* compared to other care units in the sample.
**Context variables measured with the Alberta Context Tool (ACT)**	
Leadership	A care unit was assigned a value of 1 if the proportion of care aides reporting ACT-Leadership > = 4^a^ was equal to or greater than the upper quartile among all care units in the sample. This means that the care-unit-level ACT-Leadership is *very high* compared to other care units in the sample. A care unit was assigned a value of 0 if the proportion of care aides reporting ACT-Leadership > = 4^a^ was less than the upper quartile among all care units in the sample. This means that the care-unit-level ACT-Leadership is *not very high* compared to other care units in the sample.
Social capital	A care unit was assigned a value of 1 if the proportion of care aides reporting ACT-Social Capital > = 4^a^ was equal to or greater than the upper quartile among all care units in the sample. This means that the care-unit-level ACT-Social Capital is *very high* compared to other care units in the sample. A care unit was assigned a value of 0 if the proportion of care aides reporting ACT-Social Capital > = 4^a^ was less than the upper quartile among all care units in the sample. This means that the care-unit-level ACT-Social Capital is *not very high* compared to other care units in the sample.
Organizational slack (OS) in space	A care unit was assigned a value of 1 if the proportion of care aides reporting ACT-OS Space > = 4^a^ was equal to or greater than the upper quartile among all care units in the sample. This means that the care-unit-level ACT-OS Space is *very high* compared to other care units in the sample. A care unit was assigned a value of 0 if the proportion of care aides reporting ACT-OS Space > = 4^a^ was less than the upper quartile among all care units in the sample. This means that the care-unit-level ACT-OS Space is *not very high* compared to other care units in the sample.
Organizational slack (OS) in Time	A care unit was assigned a value of 1 if the proportion of care aides reporting ACT-OS Time > = 3.25 ^a^ was equal to or greater than the upper quartile among all care units in the sample. This means that the care-unit-level ACT-OS Time is *very high* compared to other care units in the sample. A care unit was assigned a value of 0 if the proportion of care aides reporting ACT-OS Time > = 3.25^a^ was less than the upper quartile among all care units in the sample. This means that the care-unit-level ACT-OS Time is *not very high* compared to other care units in the sample.
Access to educational material	A care unit was assigned a value of 1 if the proportion of care aides who frequently or always used policy, procedure manuals, or clinical guidelines was equal to or greater than the upper quartile among all care units in the sample. This means that the care-unit-level access to educational material is *very high* compared to other care units in the sample. A care unit was assigned a value of 0 if the proportion of care aides who frequently or always used policy, procedure manuals, or clinical guidelines was less than the upper quartile among all care units in the sample. This means that the care-unit-level access to educational material is *not very high* compared to other care units in the sample.
Communication with dedicated facilitator roles	A care unit was assigned a value of 1 if the proportion of care aides who frequently or always talked with educators, clinical specialists, or someone bringing new ideas to the care unit was equal to or greater than the median among all care units in the sample. This means that the care-unit-level communication with dedicated facilitator roles is *high* compared to other care units in the sample. A care unit was assigned a value of 0 if the proportion of care aides who frequently or always talked with educators, clinical specialists, or someone bringing new ideas to the care unit was less than the median among all care units in the sample. This means that the care-unit-level communication with dedicated facilitator roles is *low* compared to other care units in the sample.
Formal meetings about care	A care unit was assigned a value of 1 if the proportion of care aides who frequently or always participated in team meetings or family conferences was equal to or greater than the median among all care units in the sample. This means that the care-unit-level formal meetings about care is *high* compared to other care units in the sample. A care unit was assigned a value of 0 if the proportion of care aides who frequently or always participated in team meetings or family conferences was less than the median among all care units in the sample. This means that the care-unit-level formal meetings about care is *low* compared to other care units in the sample.

^a^The threshold (e.g., CRU > = 4) is the median of care-aide-reported scores on a 5-level scale (1–5) in the care aide sample

#### Qualitative analysis

We used directed content analysis to examine qualitative data [56]. *A priori* codes were derived from both the PARIHS framework and ACT, encompassing elements of context that potentially influence implementation of best practices. Upon consensus of codes and definitions, 2 PhD-prepared coders coded each of the interview transcripts and fieldnotes independently using ATLAS.ti software. Both types of data were treated the same analytically. The coding team met every week to discuss coding questions and resolve coding disagreements. The coding team kept memos about rationale for coding choices and notes to clarify coding decisions. The phase of theme generation, which includes the identification of subthemes, was facilitated by two senior qualitative research experts. Themes and subthemes were derived from the coding results and were collaboratively developed during regular meetings between the two coders and the two senior qualitative research experts. These discussions continued until consensus was achieved on the themes and subthemes, and their interpretations.

### Integration of configurational and qualitative findings

We integrated findings from the coincidence analysis and qualitative insights at the stages of reporting and interpretation. We used themes (subthemes) and relevant quotes generated from the qualitative analysis to help understand contextual configurations identified in the coincidence analysis.

## Results

Table 4 contains the characteristics of care units included in our coincidence analysis (*n* = 47) and characteristics of the 3 LTC homes in the qualitative case study.

**Table 4:** Care unit characteristics and 3 case study LTC homes

Care units included in coincidence analysis (*n* = 47)					
Characteristics	n			%	
Size of LTC homes that care units are situated in					
Small (< 80 beds)	11			23.40	
Medium (80–120 beds)	7			14.89	
Large (> 120 beds)	29			61.70	
Owner-operator model of LTC homes that care units are situated in					
Public not for profit	15			31.91	
Private for profit	11			23.40	
Voluntary not for profit	21			44.68	
Province					
Alberta	28			59.57	
Saskatchewan	14			29.79	
Manitoba	5			10.64	
Unit type					
General LTC unit	26			55.32	
Secure dementia unit	21			44.68	
	Mean	SD		Range	
# of care aides per unit participating in the TREC survey	15.57	4.94		9–26	
**LTC homes included in the qualitative case study**					
	Home A		Home B		Home C
Location	Alberta		Manitoba		Saskatchewan
Number of units	6		2		2
Number of beds	228		69		100
Availability of dementia unit(s)	Yes		No		Yes
Staff participated in the qualitative interview					
Care aides	10		10		11
Registered nurse (RN)	6		3		3
Licensed practical nurse (LPN)	5		4		2
Specialist/allied personnel	11		4		2
Care manager	4		3		0
Director of care/administrators	1		1		3
Other staff (e.g., chaplain, educator)	0		5		0

### Coincidence analysis results

Our primary model of coincidence analysis identified contextual configurations linked to care units having *very high* CRU, while our secondary coincidence analysis identified contextual configurations linked to *very low* CRU. The final analytic sample comprised 47 care units (22 with *very high* CRU, 25 with *very low* CRU).

The coincidence analysis model for the outcome of *very high* CRU comprised 3 paths (i.e., minimally sufficient conditions for occurrence of the outcome), suggesting that care units had *very high* CRU (as compared to care units with *very low* CRU) if and only if they matched any of the following 3 paths (see Table 5):

**Table 5:** Summary of coincidence analysis results for the outcome of very high CRU

Context elements			Path 1	Path 2	Path 3
Leadership		Very high	**•**		
Educational material		Very high		**•**	
Social capital		Very high			**•**
Communication with dedicated facilitator roles		High			**•**
Path consistency			0.923	1.00	1.00
Path coverage			0.545	0.591	0.455
# care units falling in the path			12	13	10
Overall model	Consistency		0.95		
	Coverage		0.91		
	# unique care units covered		20		
	Total # care units with very high CRU		22		

Additional File 4 displays the analytic dataset containing the outcome and context variables included for the final coincidence analysis

Path 1: *very high* leadership.

OR

Path 2: *very high* educational material.

OR

Path 3: *very high* social capital AND *high* communication with dedicated facilitator roles.

Coverage of the final model for the outcome of *very high* CRU was 0.91, meaning that the model (containing 3 paths) covered 91% of care units (20 out of 22) that had *very high* CRU. Among 20 care units covered by the model, 9 care units exhibited only 1 path, 7 care units exhibited 2 paths, and 4 care units exhibited all 3 paths. Consistency (0.95) meant that 95% of care units exhibiting any of the 3 paths had *very high* CRU.

Modeling the outcome of *very low* CRU suggested that care units had *very low* CRU (as compared to those with *very high* CRU) if and only if they showed either of the following 2 paths (see Table 6):

**Table 6:** Summary of coincidence analysis results for the outcome of very low CRU

Context elements		Path 1	Path 2
Leadership	Not very high	•	
Organizational slack in space	Not very high	•	
Educational material	Not very high	•	
Social capital	Not very high		•
Organizational slack in time	Not very high		•
Formal meetings about care	Low		•
Path consistency		0.947	0.95
Path coverage		0.72	0.76
# care units falling in the path		18	19
Overall model	Consistency	0.92	
	Coverage	0.88	
	# unique care units covered	22	
	Total # care units with very low CRU	25	

Additional File 4 displays the analytic dataset containing the outcome and context variables included for the final coincidence analysis

Path 1: *not very high* leadership AND *not very high* organizational slack in space AND *not very high* educational material

OR

Path 2: *not very high* social capital AND *not very high* organizational slack in time AND *not very high* formal meetings about care.

Model coverage for the outcome of *very low* CRU (0.88) meant that the model (containing 2 paths) covered 88% of care units (22 out of 25) that had *very low* CRU. Among 22 care units covered by the model, 7 units exhibited only 1 path and 15 exhibited both paths. Consistency (0.92) meant that 92% of care units exhibiting any of the 2 paths had *very low* CRU.

### Qualitative results

The qualitative results identified context-related facilitators and barriers for best practice use. Data described best practice as incorporating evidence-based knowledge obtained from education, training, discussions with other staff, and other sources into routine decision-making about care. This interpretation reflected conceptual use of best practices, which aligned with what was measured in the quantitative survey. However, our qualitative data were somewhat limited in explaining contextual configurations. In the following section, we present qualitative themes about context-related facilitators and barriers, as well as qualitative insights into contextual configurations.

### Context-related facilitators

#### Leadership

Descriptions of leadership included supportive, adaptive, and empowering behaviors from both frontline (e.g., care aides’ supervisors) and middle management (e.g., unit managers) levels. In each participating home, staff reported that they overcame challenges despite contextual barriers because their leaders encouraged adaptive work (e.g., adjusting practices, resources, and behaviors in response to changing conditions and obstacles) and empowered staff to actively participate in change initiatives. Leadership also set an example by using best practices. One care aide with 11 years of experience believed that her supervisor’s strict adherence to health and safety rules benefited both residents and staff, saying, “. . very good and very precise. . she’s very strict on health and safety for the residents and for the staff.” The qualitative finding that supportive, adaptive, and empowering leadership played an overarching role in influencing best practice use, somewhat aligned with Path 1 to *very high* CRU, identified in coincidence analysis where very high leadership alone formed a path leading to *very high* CRU.

#### Social capital

Social capital was characterized as strong connections among team members that promote teamwork and effective information sharing. Care aides in all 3 case study homes stressed their reliance on other staff members and frequently used the phrase “working together” to describe their relationships with colleagues who assisted them with physically challenging tasks or other resident-related issues. Also highlighted was the critical role of communication among care aides, as well as between care aides and nurses (LPNs and RNs) in ensuring safe and quality care. For instance, care aides noted that they could avoid workplace injuries by seeking help from peers and learning from each other about residents’ behaviors to better anticipate potential problems. One care aide expressed that timely communication about resident conditions was essential when caring for residents with dementia who often exhibited behavioral reactions, stating, “We have to communicate. We’ve got good communication. Otherwise, we wouldn’t make it.”

#### Dedicated educators

All 3 homes emphasized the significance of dedicated educators and strategies to facilitate dissemination of new knowledge, to encourage changes, and to foster professional development among staff. Each home designated specific educators responsible for bringing new knowledge to the home and improving resident outcomes. In one home, a best practice leader was appointed to stay current on new research that could be implemented. Another home arranged monthly visits by a specialist to discuss fall prevention strategies with care staff and offered educational sessions for staff to consult with dementia behavior professionals. In the third home, the educator conducted an “education blitz” periodically, providing refresher education for care staff. Staff expressed appreciation for receiving new information on care practices through ongoing education sessions and communication with educators, which enabled them to use the latest development in best practices.

### Context-related barriers

#### Limited opportunities for formal communication

One significant context-related barrier to best practice use was identified as the exclusion of frontline staff, such as care aides, RNs, and LPNs, from formal meetings where important discussion and decision-making about care occurred. In one home, formal meetings to plan for the implementation of a new *gentle care relationship* model did not include frontline staff, even though they were to be key players in the success of the model. The implementation was imposed top down with minimal communication about its potential impact on frontline staff, leading to misunderstandings and a lack of motivation during the implementation process.

#### Lack of a dedicated space for discussion

Space constraints hindered seamless interaction among the care team. For example, one home lacked adequate space to have brief, yet crucial discussions about daily tasks, resulting in constant disruptions and the inability to conduct private conversations about resident care. This created a barrier to effective information sharing and connection among team members, ultimately affecting the implementation of best practices.

#### Delayed response from management

Care aides reported that delayed responses from management in addressing their questions and concerns about care acted as a barrier to using best practices. They expressed difficulties in communicating workplace issues upward, with management either not addressing the situation or taking a long time to make decisions. A care aide stated: “When decisions have to be made by management, things here just do not happen. Like they take forever. They go from person to person. Sometimes it just seems like it can go on forever, decisions with anything.”

#### Lack of time

Lack of time emerged as a significant barrier to using best practices. Care aides expressed that the high level of competing demands in their work often resulted in limited time to reflect on care tasks. They reported not having sufficient time with residents and their families to offer emotional support due to staffing shortages and inefficient scheduling systems. A care aide stated: “You get the work done, but you’re doing it as fast as you can because you have to get it done before you go home. . that can create a lot of stress and a heavy burden [laughs] because you’re rushing so—and they pay lots of money to stay here. You shouldn’t have to, you know, rush around.”

RNs also expressed concerns about struggling to follow established guidelines, protocols, or procedures due to insufficient time and staffing. A RN stated: “We have behavior management consultant people that we’ve had come in, the ideas that they gave us were a lot like the things that we already tried or if we could, if we had people to do those things, it would be great.”

Although time constraints were generally described as a significant barrier to best practice use by all staff groups and across the 3 homes, we also observed that the negative impact of time constraints was mitigated by some positive context features, including effective teamwork and timely feedback on team performance. For instance, care aides in one home shared that they saved time by working together cohesively to achieve daily goals. The interrelationship between time and teamwork or feedback observed in the qualitative data was somewhat reflected in Path 2 to *very low* CRU, which represents a combination of *lack of time*, *low social capital*, and *limited opportunities for formal communication* leading to *very low* CRU.

## Discussion

Our study enhances understanding of how various elements of organizational context may interrelate to influence best practice use in LTC settings. We focused on conceptual use of best practices with an emphasis on care aides’ use of best practices. Conceptual use of best practices is an important knowledge translation outcome that reflects individuals’ critical and reflective thinking related to using research-based knowledge in care decision-making [37–39]. We identified 3 distinct paths (contextual configurations) leading to very high levels of best practice use and another 2 paths leading to very low levels. The qualitative analysis offered additional insights into how certain context elements may act as barriers or facilitators to best practice use. It also helped explain interrelationships between context elements in 2 of the paths identified in coincidence analysis. Specifically, qualitative findings about the primacy of leadership and interrelationship of time resources with other context elements were consistent with coincidence analysis findings.

### Importance of leadership to best practice use

Our findings suggest that supportive, adaptive, and empowering leadership is sufficient to contribute to exceptional best practice use, regardless of other context-related barriers. This aligns well with a study, by Mekki et al., which proposed that transformational leadership exerts the most significant influence on implementation practices while other favorable contextual conditions only make a difference when leadership assumes an active role [19]. Transformational leadership—emphasizing long-term goals, clear roles, teamwork, learning culture, and relationship building—has been recognized as an essential context element for best practice use [7, 9, 11, 57–60]. Although we did not use an instrument designed specifically to measure transformational leadership, the ACT within the survey measured some features, specifically emotionally intelligent leadership and resonant leadership (i.e., focused on actively supporting, coaching, motivating, and relationship building with their staff) of frontline leaders (to whom care aides reported most of the time, which could be RNs, LPNs, or unit managers). Our qualitative analysis identified characteristics that align with transformational leadership, such as empowering and adaptive leadership at frontline and middle management levels.

Though we identified the primacy of leadership, the reasons behind its extensive influence on best practice use remain unclear. Future research could explore the mechanisms by which leadership influences implementation practices in LTC settings, as there may be unidentified causal chains. For example, a systematic review by Li et al. found that leadership specifically affects the use of champions, resource allocation, monitoring of feedback mechanisms, and organizational culture—all of which subsequently affect implementation practices [11]. A qualitative study spanning four countries found that nursing leaders, whether in formal management or more enabling positions, actively promoted evidence-based practice to frontline staff at the point of care through various strategies [61, 62]. Their approaches, consistent across diverse health care settings, emphasized the importance of informal relationships, role modeling, methods to verify evidence, and the cultivation and maintenance of positive working relationships [61, 62]. 

### Role of codified knowledge in best practice use

Frequent use of educational material is another sufficient condition that led to very high best practice use, based on the findings from our coincidence analysis. Educational material (e.g., policy or procedure manuals, clinical practice guidelines) contains formalized, codified knowledge about resident care. Our qualitative data did not reveal staff experiences with using those formalized education materials, limiting our ability to understand the processes by which codified knowledge facilitates care aides to internalize knowledge and develop their own cognitive frameworks for decision-making. There is limited research addressing these questions, especially within LTC settings and with a focus on care aides [1, 17, 20]. 

Our findings underscore the importance of clarity on different types of best practice use (e.g., instrumental vs. conceptual) and differentiating them [30, 37–40]. While instrumental use of best practices involves the more concrete application of codified and explicit evidence-based knowledge, conceptual use reflects an individual’s cognitive awareness, internal acceptance, and capacity to use knowledge acquired from multiple sources [30, 37–40]. In LTC homes, conceptual use of best practices is particularly important, as use of knowledge by all staff, especially unregulated staff, involves using both codified knowledge (often embedded in “rules”) and tacit knowledge relating to resident needs, making sense of their practices and responding to contextual uncertainties, rather than applying a standardized protocol [63]. 

### Dedicated facilitators and social capital

Social capital and communication with dedicated facilitator roles (e.g., clinical educators, specialists, or people bringing new ideas) must co-present to contribute to best practice use. Our qualitative findings shed light on the essential facilitator role of clinical educators in the implementation of best practices. These individuals facilitated best practice use by providing specialized training, guidance, and motivation to individuals and teams in understanding and applying best practices. However, communication between care aides and dedicated facilitator roles like clinical educators, while necessary, is not sufficient by itself to ensure high use of best practices. Some researchers have started to explore the interactive relationships between the availability of clinical educators and other context elements in affecting implementation outcomes [27], with some studies using regression-based interaction analysis [12, 13]. These studies suggest that the influence of clinical educators on best practice use is contingent on other context elements (e.g., leadership, culture, feedback process) and their interactions may exert complementary, synergistic, or buffering effects on this outcome [12, 13, 20, 64]. These findings also suggest that it may be possible to identify care units or LTC homes to which deploying scarce educator resources might be most effective [12, 13, 20, 64]. 

Our study supports existing literature which underscores the importance of social capital—reflected in effective communication and teamwork within and between care teams—in the process of using best practices [63, 65, 66]. Importantly, we specifically noted that social capital had to coexist with regular communication between care aides and clinical educators, or other dedicated facilitator roles, to enhance best practice use. Several qualitative studies in LTC homes have suggested that care aides acquire best practice knowledge through their interactions with interdisciplinary team members, such as clinical educators [63, 65, 66]. The knowledge they gain can be effectively disseminated within the care team when peer relationships are supportive and collaborative, leading to continual team improvement in the application of best practices [63, 65, 66]. Conversely, strong social capital without infusion of new knowledge can impede best practice use [63, 65, 66]. According to our survey data, a limited number of care aides on a care unit frequently interacted with clinical educators or specialists. In fact, over half of the care units had less than 14% of care aides engaging regularly with these clinical educators or specialists. However, even this small proportion could notably enhance best practice use, provided that social capital was sufficiently high.

#### Contextual configurations impeding best practice use

A combination of 3 types of context elements, when in unfavorable conditions, impeded best practice use: influence and communication (i.e., leadership or social capital), resource elements (i.e., organizational slack in space or in time), and formal opportunities for learning knowledge (i.e., frequent access to educational material or participation in formal meetings). However, our approach to creating binary context variables—with a cut-off at the higher quartile—led to limited data diversity among unfavorable context cases. This made it difficult to distinguish care units with very low context from those with generally low context, resulting in overlapping cases where many care units exhibited both paths to low best practice use. Future studies may delineate varying degrees of contextual conditions and their subsequent influence on best practice use.

Our qualitative findings shed light on the combined effects of time resources, teamwork, and feedback, indicating that the detrimental effects of time constraints on best practice use can be mitigated when the other two context elements are favorable. In the literature, time constraints due to an imbalance between job resources (e.g., staffing) and demands (e.g., complex care needs) have been consistently recognized as a significant barrier to implementing best practices and improving the quality of care [63, 65–69]. Researchers have further suggested that the negative influence of time constraints can be buffered by cohesive teams, trusting work relations, collegial communication processes, and uninterrupted information flow [63, 65, 66, 68, 69]. 

### Limitations

There are several limitations to our study which suggest the importance of further investigation. First, aggregating individual data to the care unit level may cause loss of information and reduced data variance, posing challenges for configurational analysis, which requires data diversity (i.e., cases with distinguishable conditions). Aggregating data could potentially introduce an ecological fallacy. Relationships observed at the care unit level do not necessarily reflect relationships between care aide-perceived context and best practice use at the individual level.

Another limitation lies in the mismatched levels of analysis between quantitative and qualitative data. While qualitative case study data captured observations within care units and participants’ experiences with their immediate work environment, cases in the qualitative study were initially defined as LTC homes, without distinguishing individual care units within each home. This discrepancy limited our ability to directly compare qualitative and quantitative cases at the same level of analysis, as we were unable to map individual care units within the three LTC homes participating in the case study onto the sample of care units included in the coincidence analysis This hindered the integration of coincidence analysis and qualitative analysis at an earlier stage of data analysis. Despite these challenges, the congruent conceptualization of context-related factors and the outcome—the use of best practices—permitted the eventual merging of both methods during the later stages of interpreting results. We were able to use qualitative insights across the three cases to broadly interpret the coincidence analysis results, although case-specific interpretation was not feasible.

#### Implications for future research

Beyond demonstrating the potential afforded by mixing configurational and qualitative analysis [70], this study offers important implications for future studies aiming to explore the influence of context on implementation or quality of care outcomes. Configurational analysis quantifies case-based explanations of different combinations of context elements leading to positive or negative levels of the outcome. This holistic case-based perspective mirrors the complexity of the real world situations, resulting in practically useful findings [23, 44]. LTC homes could map their contextual strengths and weaknesses within identified configurations and address specific gaps.

Our qualitative findings provide understanding of the roles of individual context elements. However, these data, initially collected for other research purposes, were limited in their ability to explain contextual configurations. Future mixed methods research could benefit from primary data collection and early integration of diverse methods during both the data collection and analysis stages to optimize the explanatory power of the mixed methods approach [70]. Additionally, it may be fruitful to mix traditional regression-based quantitative analysis (a statistical approach), configurational analysis (a mathematical approach), and qualitative analysis. Such an innovative 3-way mixed methods design has proven useful in organizational research for studying complex phenomena [31]. 

While the age of data may be seen as a limitation, our findings maintain significant relevance to the intent of this study—to optimize use of high quality secondary quantitative and qualitative data related to LTC settings through a novel mixed methods approach. Our study focused on conceptual use of best practice knowledge in everyday care decision-making instead of on implementation of specific evidence-based interventions or innovations. This presents an important opportunity for future research, which could examine context and its relationship with implementation outcomes related to specific evidence-based interventions in LTC settings using a combination of multiple methods.

## Conclusions

This study deepens our understanding of the complex interactions between contextual elements and their effects on implementing best practices in LTC homes. It also demonstrates the potential of mixing configurational and qualitative analysis to study such complex relationships, and provides important implications for futures studies aiming to use this mixed methods approach.

### Supplementary Information

Supplementary Material 1.

## Data Availability

The authors have included the final analytic data for coincidence analysis in Additional File 4. The data contains 47 care units as the analytic cases (de-identified), with both the outcome and context variables derived from original data sources through processes of aggregation and recoding. The original Translating Research in Elder Care (TREC) data used for this article are housed in the secure and confidential Health Research Data Repository (HRDR) in the Faculty of Nursing at the University of Alberta, in accordance with the health privacy legislation of participating TREC jurisdictions. The data were provided under specific data sharing agreements only for approved use by TREC within the HRDR. Where necessary, access to the HRDR to review the original source data may be granted to those who meet pre-specified criteria for confidential access, available at request from the TREC data unit.

## Abbreviations

LTC: Long-term care
PARIHS: The Promoting Action on Research Implementation in Health Services framework
CFIR: The Consolidated Framework for Implementation Research
TREC: The Translating Research in Elder Care program
RN: Registered nurses
LPN: Licensed practical nurses
CRU: Conceptual research use
ACT: The Alberta Context Tool

## References

[1] Diehl H, Graverholt B, Espehaug B, Lund H (2016). Implementing guidelines in nursing homes: a systematic review. BMC Health Serv Res.

[2] McArthur C, Bai Y, Hewston P, Giangregorio L, Straus S, Papaioannou A (2021). Barriers and facilitators to implementing evidence-based guidelines in long-term care: a qualitative evidence synthesis. Implement Sci.

[3] Boström A-M, Slaughter SE, Chojecki D, Estabrooks CA (2012). What do we know about knowledge translation in the care of older adults? A scoping review. J Am Med Dir Assoc.

[4] Zimmerman S, Sloane PD, Rashik MI (2022). Let’s rename nursing assistants what they are: professional caregivers. J Am Med Dir Assoc.

[5] Berta W, Laporte A, Deber R, Baumann A, Gamble B (2013). The evolving role of health care aides in the long-term care and home and community care sectors in Canada. Hum Resour Health.

[6] Hewko SJ, Cooper SL, Huynh H, Spiwek TL, Carleton HL, Reid S (2015). Invisible no more: a scoping review of the health care aide workforce literature. BMC Nurs.

[7] Kitson A, Harvey G, McCormack B (1998). Enabling the implementation of evidence based practice: a conceptual framework. BMJ Qual Saf.

[8] Nilsen P, Bernhardsson S (2019). Context matters in implementation science: a scoping review of determinant frameworks that describe contextual determinants for implementation outcomes. BMC Health Serv Res.

[9] Harvey G, Kitson A (2015). PARIHS revisited: from heuristic to integrated framework for the successful implementation of knowledge into practice. Implement Sci.

[10] Damschroder LJ, Aron DC, Keith RE, Kirsh SR, Alexander JA, Lowery JC (2009). Fostering implementation of health services research findings into practice: a consolidated framework for advancing implementation science. Implement Sci.

[11] Li S-A, Jeffs L, Barwick M, Stevens B (2018). Organizational contextual features that influence the implementation of evidence-based practices across healthcare settings: a systematic integrative review. Syst Reviews.

[12] Lo T, Hoben M, Norton PG, Teare GF, Estabrooks CA (2018). Importance of clinical educators to research use and suggestions for better efficiency and effectiveness: results of a cross-sectional survey of care aides in Canadian long-term care facilities. BMJ open.

[13] Lo T, Boamah SA, Poss JW, Teare GF, Norton PG, Estabrooks CA (2021). How does the Facilitation effort of clinical educators interact with aspects of Organizational Context to Affect Research Use in Long-Term Care? Evidence from CHAID Analysis. J Nurs Scholarsh.

[14] Berta W, Teare GF, Gilbart E, Ginsburg LS, Lemieux-Charles L, Davis D (2005). The contingencies of organizational learning in long-term care: factors that affect innovation adoption. Health Care Manage Rev.

[15] Dryden-Palmer K, Parshuram C, Berta W (2020). Context, complexity and process in the implementation of evidence-based innovation: a realist informed review. BMC Health Serv Res.

[16] Gagliardi AR, Webster F, Brouwers MC, Baxter NN, Finelli A, Gallinger S (2014). How does context influence collaborative decision-making for health services planning, delivery and evaluation?. BMC Health Serv Res.

[17] Rycroft-Malone J, Seers K, Chandler J, Hawkes CA, Crichton N, Allen C (2013). The role of evidence, context, and facilitation in an implementation trial: implications for the development of the PARIHS framework. Implement Sci.

[18] Westergren A (2012). Action-Oriented Study Circles Facilitate Efforts in Nursing Homes to Go from Feeding to Serving: Conceptual Perspectives on Knowledge Translation and Workplace Learning. J Aging Res.

[19] Mekki TE, Øye C, Kristensen B, Dahl H, Haaland A, Nordin KA (2017). The inter-play between facilitation and context in the promoting action on research implementation in health services framework: a qualitative exploratory implementation study embedded in a cluster randomized controlled trial to reduce restraint in nursing homes. J Adv Nurs.

[20] McCullough MB, Chou AF, Solomon JL, Petrakis BA, Kim B, Park AM (2015). The interplay of contextual elements in implementation: an ethnographic case study. BMC Health Serv Res.

[21] Cammer A, Morgan D, Stewart N, McGilton K, Rycroft-Malone J, Dopson S (2014). The hidden complexity of long-term care: how context mediates knowledge translation and use of best practices. Gerontologist.

[22] Watts AS, Vidoni ED, Loskutova N, Johnson DK, Burns JM (2013). Measuring physical activity in older adults with and without early stage Alzheimer’s Disease. Clin Gerontologist.

[23] Anderson RA, Issel LM, McDaniel RR (2003). Nursing homes as complex adaptive systems: relationship between management practice and resident outcomes. Nurs Res.

[24] Mills T, Lawton R, Sheard L (2019). Advancing complexity science in healthcare research: the logic of logic models. BMC Med Res Methodol..

[25] Pfadenhauer LM, Gerhardus A, Mozygemba K, Lysdahl KB, Booth A, Hofmann B (2017). Making sense of complexity in context and implementation: the context and implementation of Complex interventions (CICI) framework. Implement Sci.

[26] Leykum LK, Lanham HJ, Pugh JA, Parchman M, Anderson RA, Crabtree BF (2014). Manifestations and implications of uncertainty for improving healthcare systems: an analysis of observational and interventional studies grounded in complexity science. Implement Sci.

[27] Duan Y, Iaconi A, Wang J, Perez JS, Song Y, Chamberlain SA (2022). Conceptual and relational advances of the PARIHS and i-PARIHS frameworks over the last decade: a critical interpretive synthesis. Implement Sci.

[28] Kislov R, Pope C, Martin GP, Wilson PM (2019). Harnessing the power of theorising in implementation science. Implement Sci.

[29] Nilsen P (2020). Making sense of implementation theories, models, and frameworks. Implement Sci.

[30] Squires JE, Estabrooks CA, Scott SD, Cummings GG, Hayduk L, Kang SH (2013). The influence of organizational context on the use of research by nurses in Canadian pediatric hospitals. BMC Health Serv Res.

[31] Kahwati LC, Kane HL (2018). Qualitative comparative analysis in mixed methods research and evaluation.

[32] Cragun D, Nilsen P, Birken SA (2020). Configurational comparative methods. Handbook on Implementation Science.

[33] Whitaker RG, Sperber N, Baumgartner M, Thiem A, Cragun D, Damschroder L (2020). Coincidence analysis: a new method for causal inference in implementation science. Implement Sci.

[34] Quintana R. Embracing complexity in social science research. Qual Quant. 2022;57:1–24.

[35] Miech EJ, Freitag MB, Evans RR, Burns JA, Wiitala WL, Annis A (2021). Facility-level conditions leading to higher reach: a configurational analysis of national VA weight management programming. BMC Health Serv Res.

[36] Damschroder LJ, Miech EJ, Freitag MB, Evans R, Burns JA, Raffa SD (2022). Facility-level program components leading to population impact: a coincidence analysis of obesity treatment options within the Veterans Health Administration. Translational Behav Med.

[37] Squires JE, Estabrooks CA, Newburn-Cook CV, Gierl M (2011). Validation of the conceptual research utilization scale: an application of the standards for educational and psychological testing in healthcare. BMC Health Serv Res.

[38] Estabrooks CA (1999). The conceptual structure of research utilization. Res Nurs Health.

[39] Stetler CB (2001). Updating the Stetler model of research utilization to facilitate evidence-based practice. Nurs Outlook.

[40] Kramer DM, Wells RP, Carlan N, Aversa T, Bigelow PP, Dixon SM (2013). Did you have an impact? A theory-based method for planning and evaluating knowledge-transfer and exchange activities in occupational health and safety. Int J Occup Saf Ergonomics: JOSE.

[41] Estabrooks C, Song Y, Anderson R, Beeber A, Berta W, Chamberlain S (2022). The influence of context on implementation and improvement: protocol for a mixed methods, secondary analyses study. JMIR Res Protocols.

[42] Levitt HM, Bamberg M, Creswell JW, Frost DM, Josselson R, Suárez-Orozco C (2018). Journal article reporting standards for qualitative primary, qualitative meta-analytic, and mixed methods research in psychology: the APA Publications and Communications Board task force report. Am Psychol.

[43] Nelson EC, Batalden PB, Huber TP, Mohr JJ, Godfrey MM, Headrick LA (2002). Microsystems in health care: part 1. Learning from high-performing front-line clinical units. Jt Comm J Qual Improv.

[44] Lanham HJ, Sturmberg JP, Martin CM (2013). A complexity science perspective of organizational behavior in clinical microsystems. Handbook of systems and complexity in health.

[45] Estabrooks CA, Morgan DG, Squires JE, Boström A-M, Slaughter SE, Cummings GG (2011). The care unit in nursing home research: evidence in support of a definition. BMC Med Res Methodol.

[46] Norton PG, Murray M, Doupe MB, Cummings GG, Poss JW, Squires JE (2014). Facility versus unit level reporting of quality indicators in nursing homes when performance monitoring is the goal. BMJ open.

[47] Estabrooks CA, Squires JE, Cummings GG, Teare GF, Norton PG (2009). Study protocol for the translating research in elder care (TREC): building context - an organizational monitoring program in long-term care project (project one). Implement Sci.

[48] Rycroft-Malone J, Dopson S, Degner L, Hutchinson AM, Morgan D, Stewart N (2009). Study protocol for the translating research in elder care (TREC): building context through case studies in long-term care project (project two). Implement Sci.

[49] Wang J, Anderson R, Perez JS, Estabrooks CA, Berta W, Lanham HJ et al. Understanding adaptive Leadership in the context of nursing homes. J Appl Gerontol. 2024:07334648241243312. 10.1177/07334648241243312 38566520

[50] Baumgartner M, Falk C. Boolean difference-making: a modern regularity theory of causation. The British Journal for the Philosophy of Science. 2023;74(1):171–197.

[51] Estabrooks CA, Squires JE, Hayduk LA, Cummings GG, Norton PG (2011). Advancing the argument for validity of the Alberta Context Tool with healthcare aides in residential long-term care. BMC Med Res Methodol.

[52] Baumgartner M, Ambühl M. cna: An R Package for Configurational Causal Inference and Modeling. 2020.

[53] Parkkinen V-P, Baumgartner M, Ambuehl M. Package ‘frscore’ Functions for Calculating Fit-Robustness of CNA-Solutions 2022 [ https://cran.r-project.org/web/packages/frscore/frscore.pdf.

[54] Yakovchenko V, Miech EJ, Chinman MJ, Chartier M, Gonzalez R, Kirchner JE (2020). Strategy configurations directly linked to higher hepatitis C virus treatment starts: an applied use of configurational comparative methods. Med Care.

[55] Rich JA, Miech EJ, Semenza DC, Corbin TJ (2022). How combinations of state firearm laws link to low firearm suicide and homicide rates: a configurational analysis. Prev Med.

[56] Hsieh H-F, Shannon SE (2005). Three approaches to qualitative content analysis. Qual Health Res.

[57] Reichenpfader U, Carlfjord S, Nilsen P (2015). Leadership in evidence-based practice: a systematic review. Leadersh Health Serv.

[58] Meza RD, Triplett NS, Woodard GS, Martin P, Khairuzzaman AN, Jamora G (2021). The relationship between first-level leadership and inner-context and implementation outcomes in behavioral health: a scoping review. Implement Sci.

[59] Gifford W, Davies B, Edwards N, Griffin P, Lybanon V (2007). Managerial leadership for nurses’ use of research evidence: an integrative review of the literature. Worldviews Evidence-Based Nurs.

[60] Clavijo-Chamorro MZ, Romero-Zarallo G, Gómez-Luque A, López-Espuela F, Sanz-Martos S, López-Medina IM (2022). Leadership as a facilitator of evidence implementation by nurse managers: a metasynthesis. West J Nurs Res.

[61] Harvey G, Gifford W, Cummings G, Kelly J, Kislov R, Kitson A (2019). Mobilising evidence to improve nursing practice: a qualitative study of leadership roles and processes in four countries. Int J Nurs Stud.

[62] Kitson AL, Harvey G, Gifford W, Hunter SC, Kelly J, Cummings GG (2021). How nursing leaders promote evidence-based practice implementation at point‐of‐care: a four‐country exploratory study. J Adv Nurs.

[63] Janes N, Sidani S, Cott C, Rappolt S (2008). Figuring it out in the moment: a theory of unregulated care providers’ knowledge utilization in dementia care settings. Worldviews evidence-based Nurs.

[64] Slaughter SE, Estabrooks CA (2013). Optimizing the mobility of residents with dementia: a pilot study promoting healthcare aide uptake of a simple mobility innovation in diverse nursing home settings. BMC Geriatr.

[65] Caspar S, Ratner PA, Phinney A, MacKinnon K (2016). The influence of Organizational systems on Information Exchange in Long-Term Care facilities: an institutional ethnography. Qual Health Res.

[66] Madden C, Clayton M, Canary HE, Towsley G, Cloyes K, Lund D (2017). Rules of performance in the nursing home: a grounded theory of nurse–CNA communication. Geriatr Nurs.

[67] Song Y, Hoben M, Norton P, Estabrooks CA (2020). Association of work environment with missed and rushed care tasks among care aides in nursing homes. JAMA Netw open.

[68] Kolanowski A, Fick D, Frazer C, Penrod J (2010). It’s about Time: Use of Nonpharmacological interventions in the nursing home. J Nurs Scholarsh.

[69] Kolanowski A, Van Haitsma K, Penrod J, Hill N, Yevchak A (2015). Wish we would have known that! Communication breakdown impedes person-centered care. Gerontologist.

[70] Rattray NA, Miech EJ, True G, Natividad D, Laws B, Frankel RM (2023). Modeling contingency in veteran community reintegration: a mixed methods approach. J Mixed Methods Res.

