# Supplementary material for: How context links to best practice use in long-term care homes: a mixed methods study
Source: Implement Sci Commun. 2024 Jun 7;5:63. doi: 10.1186/s43058-024-00600-0 (PMC11157780; doi:10.1186/s43058-024-00600-0)
Supplement: Supplementary file 1 — Supplementary Material 1. [file 43058_2024_600_MOESM1_ESM.docx]

Additional Files

# Additional File 1 Box plots for care-unit-level continuous variables


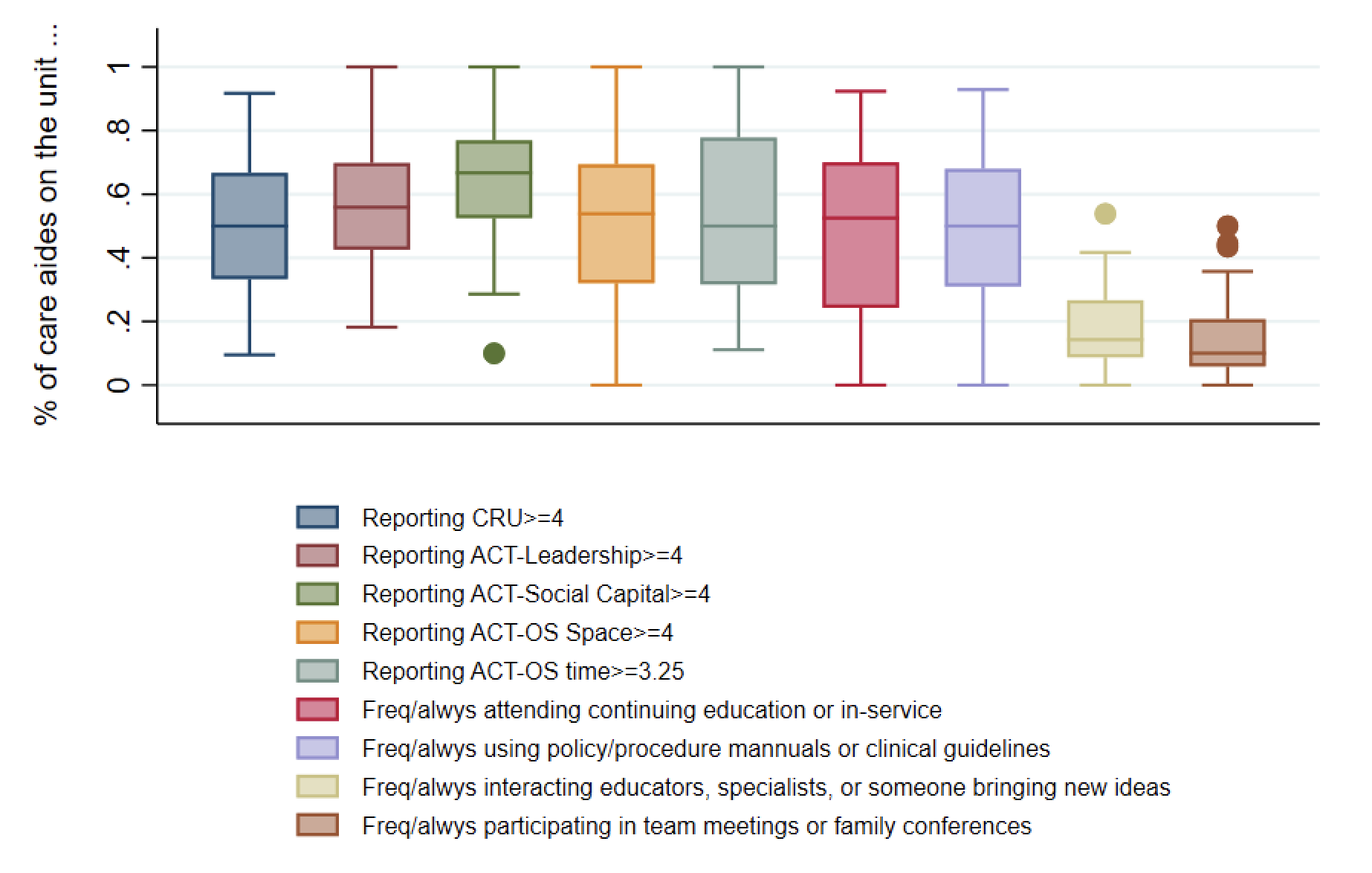


*Notes:* We aggregated individual-level, care-aide-reported scores of the original variables to the care-unit level. The aggregated care-unit-level variables were presented as continuous variables. Specifically, they were expressed as “the proportion of care aides on a given care unit that report scores for the original variables exceeding a certain threshold”. One outcome variable and eight context variables were selected for coincidence analysis. For conceptual research use, leadership, social capital, organizational slack in space, and organizational slack in time, the care-unit-level scores were expressed as “the proportion of care aides on the unit that reported the scale score equal to or above the median of care-aide-reported scores in the care aide sample”. For the four context meta-factors (each of which was derived from combing two items), the care-unit-level scores were expressed as “the proportion of care aides on the unit that reported ‘frequently’ or ‘almost always’ for any of the item”.

# Additional File 2 Description of care-unit-level categorical variables

| **Variable** | **Description and coding** | |
| --- | --- | --- |
| **Outcome:**  Conceptual research use (CRU) | 1= “<33% of care aides on the care unit reporting CRU>=4^a^”  2= “33-50% of care aides on the care unit reporting CRU>=4^a^”  3= “50-67% of care aides on the care unit reporting CRU>=4^a^”  4= “>=67% of care aides on the care unit reporting CRU>=4^a^” | We used quartile-split to categorize a given care-unit-level continuous variable into 4 levels.  The distribution of the care-unit-level continuous variable is presented in Additional File 1.  Each care-unit-level categorical variables was coded as:  1= “less than the lower quartile, meaning very low”,  2= “from lower quartile to median, meaning moderately low”,  3= “from median to upper quartile, meaning moderately high”,  4= “equal to or greater than the upper quartile, meaning very high”. |
| **Context variables measured with the Alberta Context Tool (ACT)** | |  |
| Leadership | 1= “<43% of care aides on the care unit reporting ACT-Leadership>=4^a^”  2= “43-56% of care aides on the care unit reporting ACT-Leadership>=4^a^”  3= “56-70% of care aides on the care unit reporting ACT-Leadership>=4^a^”  4= “>=70% of care aides on the care unit reporting ACT-Leadership>=4^a^” |  |
| Social capital | 1= “<52% of care aides on the care unit reporting ACT-Social Capital>=4^a^”  2= “52-67% of care aides on the care unit reporting ACT-Social Capital>=4^a^”  3= “67-77% of care aides on the care unit reporting ACT-Social Capital>=4^a^”  4= “>=77% of care aides on the care unit reporting ACT-Social Capital>=4^a^” |  |
| OS-Space | 1= “<32% of care aides on the care unit reporting ACT-OS Space>=4^a^”  2= “32-54% of care aides on the care unit reporting ACT-OS Space>=4^a^”  3= “54-69% of care aides on the care unit reporting ACT-OS Space>=4^a^”  4= “>=69% of care aides on the care unit reporting ACT-OS Space>=4^a^” |  |
| OS-Time | 1= “<32% of care aides on the care unit reporting ACT-OS Time>=3.25^a^”  2= “32-50% of care aides on the care unit reporting ACT-OS Time>=3.25^a^”  3= “50-78% of care aides on the care unit reporting ACT-OS Time>=3.25^a^”  4= “>=78% of care aides on the care unit reporting ACT-OS Time>=3.25^a^” |  |
| Educational activities | 1= “<24% of care aides on the care unit frequently or always attending continuing education out of facility or in-services in the facility”  2= “24-53% of care aides on the care unit frequently or always attending continuing education out of facility or in-services in the facility”  3= “53-70% of care aides on the care unit frequently or always attending continuing education out of facility or in-services in the facility”  4= “>=70% of care aides on the care unit frequently or always attending continuing education out of facility or in-services in the facility” |  |
| Access to educational material | 1= “<31% of care aides on the care unit frequently or always using policy/procedure manuals/clinical guidelines”  2= “31-50% of care aides on the care unit frequently or always using policy/procedure manuals/clinical guidelines”  3= “50-68% of care aides on the care unit frequently or always using policy/procedure manuals/clinical guidelines”  4= “>=68% of care aides on the care unit frequently or always using policy/procedure manuals/clinical guidelines” |  |
| Communication with dedicated facilitator roles | 1= “<14% of care aides on the care unit frequently or always interacting with educators, specialists, or someone bringing new ideas”  2= “>=14% of care aides on the care unit frequently or always interacting with educators, specialists, or someone bringing new ideas” | We used median-split to categorize a given care-unit-level continuous variable into 2 levels.  The distribution of the care-unit-level continuous variable is presented in Additional File 1.  Each care-unit-level categorical variables was coded as:  1= “less than the median, meaning low”  2= “equal to or greater than the median, meaning high” |
| Formal meetings about care | 1= “<10% of care aides on the care unit frequently or always participating in team meetings or family conferences  2= “>=10% of care aides on the care unit frequently or always participating in team meetings or family conferences |  |

*Notes.* a: The threshold (e.g., CRU>=4) is the median of care-aide-reported scores on a 5-level scale (1-5) in the care aide sample.

# Additional File 3 Sensitivity Analysis

During our case selection for coincidence analysis (**Step 3: Case Selection** in Data Calibration), we conducted a sensitivity analysis. Initially, we excluded the middle set of cases (i.e., care units that fell into the middle two categories of care-unit-level CRU: “from lower quartile to median” and “from median to upper quartile”) as reported in the manuscript. This was to ensure a substantial comparison between the very high CRU group (i.e., equal to or greater than the upper quartile) and the very low CRU group (i.e., less than the lower quartile). Such an approach is common in coincidence analysis to establish a significant difference between outcome levels. We also conducted a sensitivity check by separately modelling moderately low CRU outcomes (i.e., care units that fell into the category of “from lower quartile to median”) and moderately high CRU outcomes (i.e., care units that fell into the category of “from median to upper quartile”). This resulted in model ambiguity, making the results less interpretable and actionable, thereby justifying our initial decision to exclude the middle set of cases.

We also conducted a sensitivity analysis during the recoding of context variables (**Step 2: Refine model inputs by recalibrating variables** in Data Analysis). As reported in the manuscript, we initially recoded the quartile-split care-unit-level context variables to binary variables by grouping the three lower categories into one category (i.e., lower than the upper quartile). This was based on the results of minimally sufficient conditions. In a sensitivity check, we recoded these context variables by grouping the two lower categories into one category (i.e., less than the median) and grouping the two higher categories into one category (i.e., equal to or greater than the median). However, this led to insufficient diversity in the data required for outcome modelling, as most very high CRU cases had context scores within “equal to or greater than the median” categories, thereby validating our initial decision to group the three lower categories.

# Additional File 4 Analytic data set with the outcome and context elements included for the final coincidence analysis

| Care unit ID | Leadership | Social capital | Organizational slack in space | Organizational slack in time | Educational material | Communication with dedicated facilitator roles | Formal meetings about care | Outcome: conceptual research use |
| --- | --- | --- | --- | --- | --- | --- | --- | --- |
| A0101 | 0 | 1 | 1 | 1 | 0 | 1 | 1 | 1 |
| A0102 | 1 | 1 | 1 | 1 | 1 | 1 | 0 | 1 |
| A0103 | 0 | 1 | 1 | 1 | 0 | 1 | 1 | 1 |
| A0104 | 1 | 1 | 0 | 1 | 1 | 1 | 1 | 1 |
| A0105 | 1 | 0 | 1 | 0 | 1 | 1 | 0 | 1 |
| A0106 | 0 | 0 | 0 | 1 | 1 | 1 | 1 | 1 |
| A0301 | 0 | 0 | 0 | 1 | 1 | 0 | 0 | 1 |
| A0302 | 1 | 0 | 0 | 1 | 1 | 1 | 1 | 1 |
| A0303 | 1 | 0 | 0 | 1 | 0 | 1 | 0 | 1 |
| A0304 | 1 | 1 | 0 | 1 | 1 | 1 | 0 | 1 |
| A0401 | 1 | 1 | 0 | 0 | 0 | 1 | 0 | 1 |
| A0501 | 0 | 1 | 0 | 0 | 1 | 1 | 1 | 1 |
| A0502 | 0 | 0 | 0 | 1 | 1 | 1 | 1 | 1 |
| A0503 | 0 | 0 | 0 | 1 | 1 | 1 | 1 | 1 |
| A0601 | 1 | 0 | 0 | 1 | 1 | 1 | 1 | 1 |
| A0602 | 0 | 0 | 0 | 1 | 1 | 1 | 1 | 1 |
| A0603 | 1 | 1 | 0 | 1 | 0 | 1 | 1 | 1 |
| B0201 | 1 | 1 | 1 | 0 | 1 | 1 | 1 | 1 |
| B0203 | 1 | 1 | 0 | 0 | 0 | 1 | 0 | 1 |
| C0201 | 1 | 0 | 0 | 0 | 0 | 1 | 1 | 1 |
| C0301 | 0 | 0 | 1 | 0 | 0 | 1 | 1 | 1 |
| C0401 | 0 | 0 | 0 | 0 | 0 | 1 | 1 | 1 |
| A0201 | 1 | 1 | 0 | 0 | 0 | 0 | 0 | 0 |
| B0102 | 0 | 0 | 1 | 0 | 0 | 0 | 0 | 0 |
| B0202 | 0 | 1 | 0 | 0 | 0 | 0 | 1 | 0 |
| B0301 | 0 | 0 | 0 | 0 | 0 | 1 | 0 | 0 |
| B0401 | 0 | 0 | 0 | 0 | 0 | 1 | 0 | 0 |
| B0501 | 0 | 0 | 0 | 0 | 0 | 1 | 0 | 0 |
| B0601 | 0 | 0 | 0 | 0 | 0 | 0 | 0 | 0 |
| B0701 | 0 | 0 | 0 | 0 | 0 | 0 | 0 | 0 |
| B0801 | 0 | 0 | 0 | 0 | 0 | 0 | 0 | 0 |
| C0101 | 0 | 0 | 1 | 0 | 0 | 0 | 1 | 0 |
| C0102 | 0 | 0 | 1 | 0 | 0 | 0 | 1 | 0 |
| D0101 | 0 | 0 | 0 | 0 | 0 | 0 | 0 | 0 |
| D0102 | 0 | 0 | 0 | 0 | 0 | 0 | 0 | 0 |
| D0201 | 0 | 0 | 0 | 0 | 0 | 0 | 0 | 0 |
| D0301 | 0 | 0 | 0 | 0 | 0 | 0 | 0 | 0 |
| D0401 | 0 | 0 | 0 | 0 | 0 | 0 | 0 | 0 |
| D0501 | 0 | 0 | 1 | 0 | 0 | 0 | 0 | 0 |
| D0502 | 0 | 0 | 0 | 0 | 0 | 0 | 0 | 0 |
| D0503 | 0 | 0 | 0 | 0 | 0 | 0 | 0 | 0 |
| D0601 | 0 | 0 | 0 | 0 | 0 | 0 | 1 | 0 |
| D0701 | 0 | 0 | 1 | 0 | 0 | 0 | 0 | 0 |
| D0801 | 0 | 0 | 0 | 0 | 0 | 0 | 0 | 0 |
| D0901 | 0 | 0 | 0 | 0 | 0 | 0 | 1 | 0 |
| D1001 | 0 | 0 | 0 | 0 | 0 | 0 | 0 | 0 |
| D1002 | 0 | 0 | 1 | 0 | 0 | 0 | 0 | 0 |

# Additional File 5 Completed Checklist for the Mixed Methods Article Reporting Standards (MMARS)

Reference: Levitt, H. M., Bamberg, M., Creswell, J. W., Frost, D. M., Josselson, R., & Suárez-Orozco, C. (2018). Journal article reporting standards for qualitative primary, qualitative meta-analytic, and mixed methods research in psychology: The APA Publications and Communications Board task force report. *American Psychologist*, *73*(1), 26.

| **Paper section or element** | **Description of information to be reported** | **Guidance for Authors/Reviewers** | **Location where item**  **is reported** |
| --- | --- | --- | --- |
| **Title page** | | | |
| Title | - See the JARS–Qual and JARS–Quant Standards. | Authors:   - Refrain from using words that are either qualitative (e.g., “explore,” “understand”) or quantitative (e.g., “determinants,” “correlates”), because mixed methods stands in the middle between qualitative and quantitative research. - Reference the mixed methods, qualitative methods, and quantitative methods used. | See Title page |
| Author note | - See the JARS–Qual and JARS–Quant Standards. |  | See Declaration |
| Abstract | - See the JARS–Qual and JARS–Quant Standards. - Indicate the mixed methods design, including types of participants or data sources, analytic strategy, main results/findings, and major implications/significance. | Authors:   - Specify the type of mixed methods design used. See the note on types of designs in the Research Design Overview section of this table. - Consider using one keyword that describes the type of mixed methods design and one that describes the problem addressed. - Describe your approach(es) to inquiry and, if relevant, how intersecting approaches to inquiry are combined when this description will facilitate the review process and intelligibility of your paper. If your work is not grounded in a specific approach(es) to inquiry or your approach would be too complicated to explain in the allotted word count, however, it would not be advisable to provide explication on this point in the abstract. | See Abstract |
| **Introduction** | | | |
| Description of Research Problems/ Questions | - See the JARS–Qual and JARS–Quant Standards. | Authors:   - This section may convey barriers in the literature that suggest a need for both qualitative and quantitative data.   Reviewers:   - Theory or conceptual framework use in mixed methods varies depending on the specific mixed methods design or procedures used. Theory may be used inductively or deductively (or both) in mixed methods research. | See the Background section |
| Study Objectives/Aims/Research Goals | - See the JARS–Qual and JARS–Quant Standards. - State three types of research objectives/aims/goals: qualitative, quantitative, and mixed methods. Order these goals to reflect the type of mixed methods design used. - Describe the ways approaches to inquiry were combined, as it illuminates the objectives and mixed methods rationale (e.g., descriptive, interpretive, feminist, psychoanalytic, postpositivist, critical, postmodern, constructivist, or pragmatic approaches). | Reviewers:   - A mixed methods objective, aim, or goal may not be familiar to reviewers. It describes the results to be obtained from using the mixed methods design type where “mixing” or integration occurs (e.g., the aim is to explain quantitative survey results with qualitative interviews in an explanatory sequential design). For instance, the goal of a qualitative phase could be the development of a conceptual model, the goal of a quantitative phase could be hypothesis testing based upon that model, and the goal of the mixed methods phase could be to generate integrated support for a theory based upon quantitative and qualitative evidence. | See the “Study purpose and aims” under the Background section |
| **Methods** | | | |
| Research Design Overview | - See the JARS–Qual and JARS–Quant Standards. - Explain why mixed methods research is appropriate as a methodology given the paper’s goals. - Identify the type of mixed methods design used and define it. - Indicate the qualitative approach to inquiry and the quantitative design used within the mixed methods design type (e.g., ethnography, randomized experiment). - If multiple approaches to inquiry were combined, describe how this was done and provide a rationale (e.g., descriptive, interpretive, feminist, psychoanalytic, postpositivist, critical, postmodern, constructivist, or pragmatic approaches), as it is illuminating for the mixed method in use. - Provide a rationale or justification for the need to collect both qualitative and quantitative data and the added value of integrating the results (findings) from the two databases | Reviewers:   - Because mixed methods research is a relatively new methodology, it is helpful to provide a definition of it from a major reference in the field. - Mixed methods research involves rigorous methods, both qualitative and quantitative. Refer to the JARS–Qual standards (qualitative) and JARS–Quant standards (quantitative) for details of rigor. - One of the most widely discussed topics in the mixed methods literature would be research designs. There is not a generic mixed methods design but rather multiple types of designs. Basic, core designs include convergent design, explanatory sequential design, and exploratory sequential design. Although the names and types of designs may differ among mixed methods writers, a common understanding is that procedures for conducting a mixed methods study may differ from one project to another. Further, these basic procedures can be expanded by linking mixed methods to other designs (e.g., an intervention or experimental trial mixed methods study), to theories or standpoints (e.g., a feminist mixed methods study), or to other methodologies (e.g., a participatory action research mixed methods study). | See the “Study design” subsection under the Methods section |
| Participants or Other Data Sources | - See the JARS–Qual and JARS–Quant Standards. - When data are collected from multiple sources, clearly identify the sources of qualitative and quantitative data (e.g., participants, text), their characteristics, and the relationship between the data sets, if there is one (e.g., an embedded design). - State the data sources in the order of procedures used in the design type (e.g., qualitative sources first in an exploratory sequential design followed by quantitative sources), if a sequenced design is used in the mixed methods study. | Authors:   - Because multiple sources of data are collected, separate descriptions of samples are needed when they differ. A table of qualitative sources and quantitative sources is helpful. This table could include type of data, when data were collected, and from whom. This table might also include study aims/research questions for each data source and anticipated outcomes of the study. In mixed methods research, this table is often called an "implementation matrix." - Rather than describe data as represented in numbers versus words, it is better to describe sources of data as open-ended information (e.g., qualitative interviews) and closed-ended information (e.g., quantitative instruments). | See the “Data sources, setting, and sample” subsection under the Methods section |
| Researcher Description | - See the JARS–Qual Standards. | Reviewers:   - Because mixed methods research includes qualitative research, and reflexivity is often included in qualitative research, we recommend statements as to how the researchers’ backgrounds influence the research.   Authors:   - It is helpful to establish in a publication the researchers’ experiences (or research teams’ experiences) with both qualitative and quantitative research as a prerequisite for conducting mixed methods research. | See the “Qualitative analysis” subsection under the Methods section |
| Participant Sampling or Selection | - See the JARS–Qual and JARS–Quant Standards. - Describe the qualitative and the quantitative sampling in separate sections. - Relate the order of the sections to the procedures used in the mixed methods design type. |  | See the “Data sources, setting, and sample” subsection under the Methods section |
| Participant Recruitment | - See the JARS–Qual and JARS–Quant Standards. - Discuss the recruitment strategy for qualitative and quantitative research separately. |  | Participant recruitment is briefly outlined in the “Data sources, setting, and sample” subsection of the Methods section. As this is a secondary analysis, the protocols of the original study were referenced, offering in-depth information about recruitment |
| Data Collection/Identification Procedures | - See the JARS–Qual and JARS–Quant Standards. |  | Same as above. |
| Recording and Transforming the Data | - See the JARS–Qual Standards. |  | See the “Qualitative analysis” subsection under the Methods section |
| Data Analysis | - See the JARS–Qual and JARS–Quant Standards. - Devote separate sections to the qualitative data analysis, the quantitative data analysis, and the mixed methods analysis. This mixed methods analysis consists of ways that the quantitative and qualitative results will be “mixed” or integrated according to the type of mixed methods design used (e.g., merged in a convergent design, connected in explanatory sequential designs and in exploratory sequential designs). |  | See the “Coincidence analysis” and “Qualitative analysis” subsections under the Methods section |
| Validity, Reliability, and Methodological Integrity | - See the JARS–Qual and JARS–Quant Standards. - Indicate methodological integrity, quantitative validity and reliability, and mixed methods validity or legitimacy. Further assessments of mixed methods integrity are also indicated to show the quality of the research process and the inferences drawn from the intersection of the quantitative and qualitative data. |  | See the “Integration of configurational and qualitative findings” subsections under the Method section |
| **Findings/Results** | | | |
| Findings/Results subsections | - See the JARS–Qual and JARS–Quant Standards. - Indicate how the qualitative and quantitative results were “mixed” or integrated (e.g., discussion; tables of joint displays; graphs; data transformation in which one form of data is transformed to the other, such as qualitative text, codes, themes are transformed into quantitative counts or variables). | Authors:   - In mixed methods research, the Findings section typically includes sections on qualitative findings, quantitative results, and mixed methods results. This section should mirror the type of mixed methods design in terms of sequence (i.e., whether quantitative strand or qualitative strand comes first; if both are gathered at the same time, either qualitative findings or quantitative results could be presented first).   Reviewers:   - In mixed methods Results sections (or in the Discussion section to follow), authors are conveying their mixed methods analysis through “joint display” tables or graphs that array the qualitative results (e.g., themes) against the quantitative results (e.g., categorical or continuous data). This enables researchers to directly compare results or to see how results differ between the quantitative and qualitative strands. | See the Results section |
| **Discussion** | | | |
| Discussion subsections | - See the JARS–Qual and JARS–Quant Standards. | Authors:   - Typically, the Discussion section, like the Method and Findings/Results, mirrors in sequence the procedures used in the type of mixed methods design. It also reflects on the implications of the integrated findings from across the two methods. | See the Discussion section |
